# Impacts of different intensities of exercise on inflammation and hypoxia markers in low altitude

**DOI:** 10.1186/s13102-021-00375-0

**Published:** 2021-11-22

**Authors:** Fatih Baygutalp, Yusuf Buzdağlı, Murat Ozan, Mitat Koz, Nurcan Kılıç Baygutalp, Gökhan Atasever

**Affiliations:** 1 Department of Physical Medicine and Rehabilitation, Ataturk University Faculty of Medicine, Erzurum, Turkey; 2 Department of Physical Education and Sports, Erzurum Technical University Faculty of Sport Sciences, Erzurum, Turkey; 3 Department of Physical Education and Sports, Ataturk University Kazım Karabekir Education Faculty, Erzurum, Turkey; 4 Department of Sports Health Sciences, Ankara University Faculty of Sport Sciences, Ankara, Turkey; 5 Department of Biochemistry, Ataturk University Faculty of Pharmacy, Erzurum, Turkey; 6 Department of Recreation, Ataturk University Faculty of Sport Sciences, Erzurum, Turkey

**Keywords:** Anti-inflammatory cytokine, Exercise, Health, Hypoxia, Pro-inflammatory cytokine

## Abstract

**Background:**

This study aims to determine and compare the effects of exercise modalities with different intensities on the secretion of key inflammation and hypoxia markers in amateur athletes.

**Methods:**

Twenty-three athletes with a mean age of 20.1 years, living at low altitude (1850 m) participated in this study. The participants' maximal oxygen consumption values (VO_2_ max) were determined with an incremental cycle exercise test as 54.15 ± 6.14 mL kg min^−1^. Athletes performed four protocols: at rest, 50% VO_2_ max, 75% VO_2_ max and 100% VO_2_ max (until exhaustion) with one-week intervals. 50% VO_2_ max, 75% VO_2_ max sessions were performed continuously for 30 min on a bicycle ergometer and 100% VO_2_ max session was performed by cycling until exhaustion. Blood samples were obtained at rest and immediately after each exercise session. Serum tumor necrosis factor alpha (TNF-α), C-reactive protein (CRP), interleukin-10 (IL-10), and hypoxia inducible factor-1 alpha (HIF-1α) levels were measured.

**Results:**

There were significant differences in serum TNF-α levels in 75% VO_2_ max and 100% VO_2_ max sessions (489.03 ± 368.37 and 472.70 ± 365.21 ng/L, respectively) compared to rest conditions (331.65 ± 293.52 ng/L). Serum CRP levels of 50% VO_2_ max and 75% VO_2_ max sessions (1.19 ± 0.50; 1.07 ± 0.52 mg/L) were significantly higher than the rest condition (0.74 ± 0.35 mg/L). There were significant differences in serum IL-10 levels of rest condition and 50% VO_2_ max; 50% VO_2_ max, and 100% VO_2_ max sessions (328.09 ± 128.87; 446.36 ± 142.84; 347.44 ± 135.69; 324.88 ± 168.06 pg/mL). Serum HIF-1α levels were significantly higher in 75% VO2 max session compared to rest (1.26 ± 0.16; 1.08 ± 0.19 ng/mL) (*P* < 0.05 for all comparisons).

**Conclusions:**

Both inflammatory and anti-inflammatory pathway is induced on different exercise intensities. Exercise protocols performed until exhaustion may lead to activation of inflammatory pathways and hypoxia-induced damage.

## Background

The optimal exercise type and intensity should be carefully determined, particularly in acute exercise protocols. While moderate exercise generally improves immune function, excessive amounts of prolonged, high-intensity exercise may lead to impairments in immune function [1]. There are promising results from comparison studies demonstrating the equality or superiority of high-intensity intermittent training (HIIT) programs over low-intensity regular exercise programs based on cardiorespiratory and metabolic parameters [2–4]. For this reason, there is a growing interest in the studies with high-intensity intermittent training protocols since this training type is considered helpful for people who cannot exercise regularly, and HIIT protocols are time efficient [5, 6].

Exercise and intense training affect hormonal release, creating adaptive responses that will facilitate the organism to cope with exercise stress [7]. Further, exercise may be considered as a medicine against metabolic syndrome. The inflammatory response is the body's reaction and defence against homeostasis disorders, particularly infection and injury [8]. It has long been recognized that exercise is related to anti-inflammatory pathways [9, 10]. However, the pro-inflammatory pathway may be activated, in eccentric exercise protocols [11, 12]. Therefore, optimal exercise protocol should be used for athletes and sedentary people to improve outcomes and prevent musculoskeletal damages, cardiovascular, neurological, and endocrinological side effects [13, 14].

The release of pro-inflammatory cytokines and anti-inflammatory cytokines into the circulation in response to exercise varies according to exercise type, duration, and intensity [1, 13].

Tumor necrosis factor alpha (TNF-α) is an essential mediator of the acute inflammatory response [15]. Interleukin-10 (IL-10) is one of the most important anti-inflammatory cytokines [5]. Increased serum TNF- α and C-reactive protein (CRP) levels and decreased IL-10 levels can be regarded as typical signs of a pro-inflammatory state [9]. In addition, the IL-10/TNF-α ratio can be used as an indicator of the beneficial effects of exercise [16]. Many studies investigate the inflammatory response in exercise [11, 12] or training sessions [9, 17], and the results are inconsistent. These inconsistencies may arise from various exercise or training protocols (type, duration and intensity), blood sampling timing, lack of control group, small sample sizes, ethnicity and biological variations.

CRP is the main acute phase protein in tissue damage and other inflammatory conditions and is a sensitive and objective marker [18]. As a result of a systematic review on CRP, it was found that in trained athletes, when a single exercise protocol was applied, CRP temporarily increased as the acute phase response after exercise. In contrast, those who did higher levels of physical activity in longitudinal studies had lower CRP levels. In this context, although physical activity has been found to raise the CRP level acutely, it has been found that chronically physical activity reduces CRP levels [19].

Hypoxia is one of the stress factors that can promote the inflammation process, including pro-inflammatory and anti-inflammatory pathways. Also inflammatory tissues usually become hypoxic [20]. Hypoxia or decreased oxygen levels result in many changes at the cellular level, including mitochondrial biogenesis and angiogenesis in rest [21, 22] and exercise conditions [23]. Peroxisome proliferator-activated receptor-gamma coactivator 1 alpha (PGC-1α), hypoxia-inducible factor-1 alpha (HIF-1α) and vascular endotheial growth factor (VEGF) play key roles in these adaptation mechanisms. HIF-1α is a hypoxia-induced transcription factor that transcribes more than 100 enzymes and proteins involved in cellular responses caused by hypoxia [24].

There is a relationship between the body's response to inflammation or exercise stress and its response to hypoxia, and in both cases, the hypoxia-inducible factor-1a (HIF-1a) signaling pathway can be induced [25]. It is known that both exercise and hypoxia can alter mRNA expression and protein release of pro-and anti-inflammatory cytokines, activate lymphocytes, alter chemokine receptors, or induce other signaling pathways of the hypoxic inflammatory response [26, 27].

This study was conducted in a low altitude (1850 m) city [28], which is the highest city of Turkey. People living in altitudes higher than sea level have reduced hypoxic ventilatory response, decreased pulmonary hypertension under hypoxia, increased heart rate, and improved peripheral oxygen saturation [29]. The fact that the hypoxia and inflammatory responses were evaluated on different exercise intensities and that the study was conducted in a low altitude region is prominent in our study. For these reasons, in this study, the acute effects of exercise intensity on hypoxia and inflammatory responses in an amateur athlete group living in a low altitude region were investigated. The combined effects of hypoxia marker and exercise on inflammatory pathways were assessed.

## Methods

Twenty-three amateur male athletes (soccer) living at low altitude (1850 m), training 2 h/day, 5 days/week, were included in this study. Inclusion criteria were; to be an amateur athlete between the ages of 18–22, male gender, living in this location for at least 5 years, volunteering to participate in research being healthy and not having a chronic or acute illness. Exclusion criteria were; having any chronic disease, using any medication or stimulants, smoking and alcohol use, having limitation of movement due to injury for any reason. Twenty-three people who met the inclusion criteria were included to the study. Demographical characteristics of athletes are given in Table 1. Athletes were evaluated at rest and at three different exercise intensities: 30 min of exercise on a bicycle ergometer at 50% of the predetermined maximal oxygen consumption capacity (VO_2_ max) values, 75% of the predetermined VO_2_ max and exercise at 100% VO_2_ max-until the individual is exhausted. Venous blood samples were taken at rest state (1st session) and immediately after each exercise sessions (2nd, 3rd and 4th sessions).

**Table 1:** Demographical characteristics of athletes

	Male athletes (n = 23)	
	Mean ± SD	95% CI
Age (year)	20.12 ± 0.15	13.51–26.49
BMI (kg/m^2^)	23.44 ± 1.29	22.44–23.56
BFP	15.19 ± 7.19	11.89–18.11
Sports age (year)	10.21 ± 2.31	9.00–11.00
VO_2_ max (mL kg min^−1^)	54.15 ± 6.14	51.34–56.66

SD: standart deviation, CI: confident interval, BMI: body mass index, BFP: body fat percentage, VO_2_ max: maximal oxygen consumption

### Ethical ıssues

The informed consent form was obtained from all participants, and they were enlightened with all matters related to the study. The study was approved by the Clinical Research Ethics Committee of Ataturk University Faculty of Medicine (27.05.2021).

## Study design

**Fig. 1:**
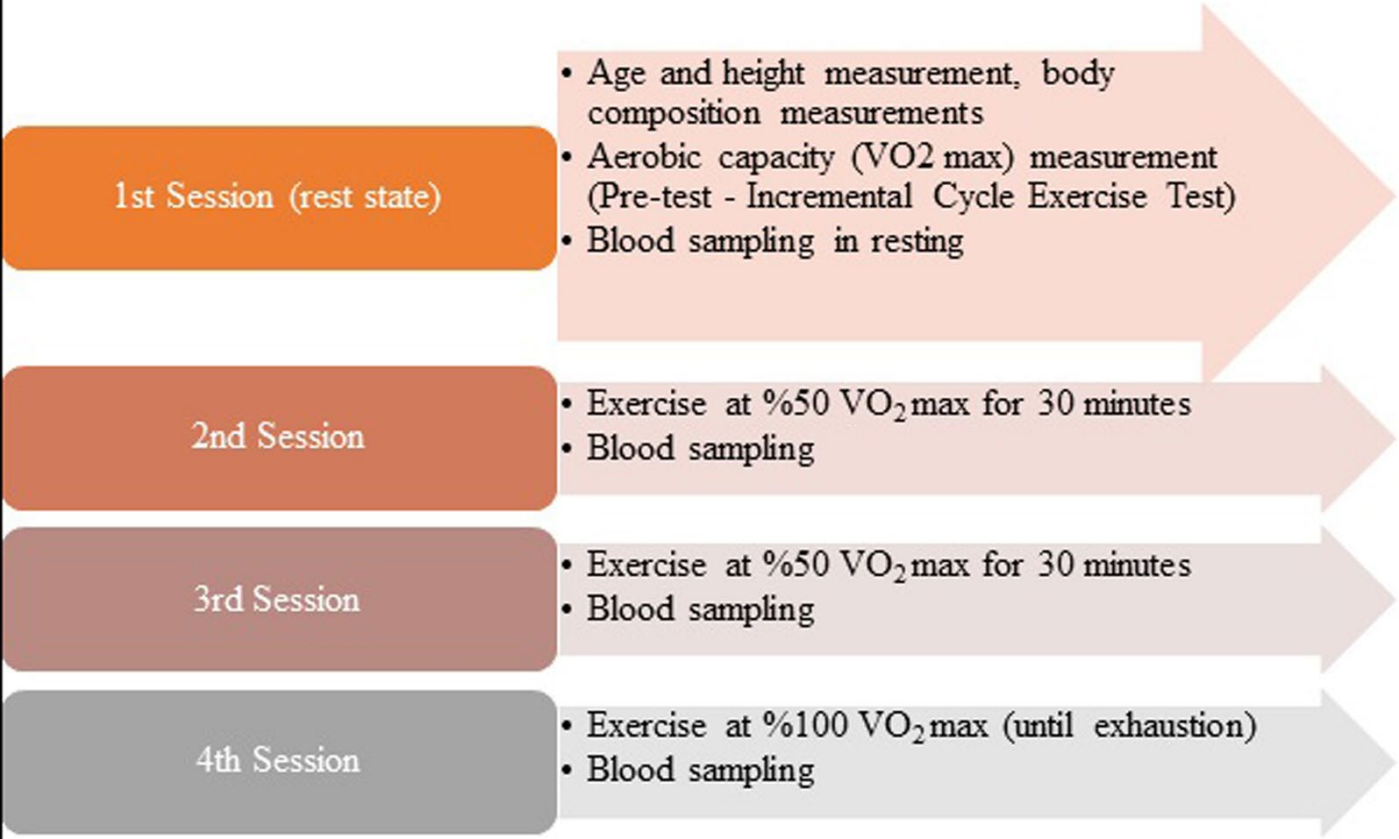
Study design

The study design is summarized in Fig. 1. 2nd session can be defined as mild intensity exercise (50% for 30 min), 3rd session as moderate-intensity exercise (75% for 30 min) and 4th session as high-intensity exercise (100% to exhaustion).

Exercise sessions were carried out at one-week intervals to prevent physiological adaptation.

### Data collection

#### Demographical characteristics and height measurement

The athletes' ages in the study were recorded based on their identity information, and the sports ages based on their declarations. The height of the athletes was measured with a mechanical measuring rod (Seca 216, Medisave UK Co., UK).

### Body composition measurement

Body composition parameters (weight and body fat percentage) were measured with the BOD POD body composition tracking system (Cosmed, USA). Body mass index (BMI) was calculated with the following formula: BMI = weight/height^2^ (kg/m^2^).

### Maximal oxygen consumption capacity (VO_2_ max) measurement (pre-test − incremental cycle exercise test)

The participants were subjected to a maximal exercise test with an exercise protocol with increasing intensity following the test completion criteria used in the bicycle ergometer protocols used for *maximal oxygen consumption* capacity and power measurements.

Participants first cycled on the bicycle ergometer for 5 min (50–60 RPM) to warm up. Then, the warming was completed by stretching for 2 min. As soon as the participant is fully ready, the test is started with the start command and the continuously increasing load test is applied. The participant started the test at 60 revolutions per minute and by pedaling at 150 watts. Then, 30 watts were increased every 2 min, and the trial continued until the test pedal speed fell below 50 revolutions per minute or the subject could not continue anymore. The Rating of Perceived Exertion (RPE) was defined with the Borg scale to determine the VO_2_ max of the participants [30]. Participant-reported Borg scale scores were used at each load-increasing phase of the exercise test in the first session they visited the laboratory and immediately after each session.

The observation of three of the criteria simultaneously was accepted to indicate that the maximal oxygen use capacity was reached, then the test was terminated. The criteria were; reporting a Borg scale score of 20 by the participant, the oxygen consumption does not increase despite the increase in workload, the ratio of carbon dioxide production to oxygen consumption RER (respiratory exchange ratio) reaches 1.15 and above, the heart rate is 85% and above the maximum number of heart rates, the increase in the number of heart rates despite the increasing workload [31]. In the gas analysis, minute ventilation (VE), oxygen volume per minute (VO_2_), produced carbon dioxide volume (VCO_2_) per minute were directly measured and recorded. At the same time, the heart rate (HRmax) at which the athletes reached the maximal oxygen use and the perceived difficulty values ​​at each step of increase were also recorded. Before the measurement sessions, the Cosmed K5 oxygen analyzer was calibrated with high-grade calibration gases provided by the manufacturers. Gas was pumped from the flow meters with a 3-L calibration syringe following the manufacturer's recommendations and heated for a minimum of 15 min. Mask size was determined individually before the first test, and measurements were taken with the same size in subsequent sessions.

### Exercise protocols

All of the test protocols were carried out in our university's Sports performance laboratory and measurement center. Athletes were not allowed to perform vigorous exercise, using drugs, caffeine, alcohol and performance-enhancing ergogenic supplements from 48 h before exercise protocols. Before starting exercise protocols (pre-test), the participants' maximal oxygen consumption values (VO_2_ max) were determined using the oxygen analyzer K5 (Cosmed, USA) as a pre-test with the gradually increasing load exercise test on the bicycle ergometer. Participants were required to cycle continuously for 30 min on a bicycle ergometer at 50% and 75% of the predetermined maximal oxygen consumption capacity values.

Finally the participants were required to cycle at 100% VO_2_max until exhaustion.The term 100% VO2 max defines the situation when the participant reaches exhaustion during exercise. [32]. The mean time to exhaustion of athletes was 16.35 ± 3.38 min. Venous blood samples were taken immediately after each session. All measurements were taken at the same time of day (morning time).

### Biochemical analysis

5 mL of venous blood samples were taken from each athlete. After the serum was obtained, the samples were aliquoted and stored at -80 C° until analysis. Serum CRP, TNF-α, interleukin-10 and HIF-1α levels in all samples were analyzed by ELISA method with commercial kits (Bioassay Technology Laboratory-BT Lab, China produced all kits). Samples were collected once and measured duplicated.

### Sample size calculation

The minimum number of patients required for the study was calculated in the G Power sample calculation program (version 3.1.9.4) at the level of Type I error (α) 0.05) and Type II error (1-β) 0.95, with an effect size (Cohen's f) of 0.4 (large) for a priori calculation of ANOVA test for 4 repeated groups. Accordingly, the minimum number of samples was determined as 16. We included 23 participants to the study, in order to prevent a limitation caused by small sample size.

### Statistical analyses

Statistical analysis was performed in SPSS 23.0 package program. Kolmogorov–Smirnov test was used to determine the normality of data. Descriptive statistical analysis, repeated measures ANOVA test, and Pearson correlation analysis were performed. Data were presented as mean ± SD (standard deviation). Kolmogorov–Smirnov test revealed that data were distributed normally, and repeated measures ANOVA test was used to compare biochemical values of different sessions. Values of *P* < 0.05 at a 95% confidence interval were considered statistically significant. Eta-squared value (η^2^) was used to determine effect sizes within the ANOVA calculation. η^2^ values of 0.01, 0.06, and 0.14 were interpreted as “small”, “medium” and “large” effect sizes, respectively.

## Results

Serum IL-10, TNF-α, CRP and HIF-1α values obtained at rest conditions and different exercise sessions are given in Table 2. Additionally, IL-10/TNF-α ratio was used as a positive predictor of exercise and presented the results in Table 2. Results show that IL-10/TNF-α ratio was decreased in 100% VO_2_ max session compared to both rest and 50% VO_2_ sessions (*P* = 0.008 and *P* = 0.041, respectively).

**Table 2:** Biochemical values of athletes

	Rest state	50% VO_2_ max	75% VO_2_ max	100% VO_2_ max	η^2^
IL-10 (pg/mL)	328.09 ± 128.87^a^	446.36 ± 142.84^d^	347.44 ± 135.69	324.88 ± 168.06	0.546
TNF-α (ng/L)	331.65 ± 293.52^b,c^	395.59 ± 319.82	472.70 ± 365.21	489.03 ± 368.37	0.309
IL-10/ TNF-α	1.63 ± 1.20 ^c^	1.49 ± 0.93^d^	1.34 ± 0.97	0.99 ± 0.67	0.566
CRP (mg/L)	0.74 ± 0.35^a,b^	1.19 ± 0.50	1.07 ± 0.52	0.97 ± 0.55	0.773
HIF-1α (ng/mL)	1.08 ± 0.19^b^	1.12 ± 0.32	1.26 ± 0.16	1.18 ± 0.21	0.453

η^2^: Eta-squared value

^a,b,c,d^Show repeated measures ANOVA Bonferroni post-hoc test *P* values

^a^Significant difference (*P* < 0.05) between rest state and 50% VO_2_ max session

^b^Significant difference (*P* < 0.05) between rest state and 75% VO_2_ max session

^c^Significant difference (*P* < 0.05) between rest state and 100% VO_2_ max session

^d^Significant difference (*P* < 0.05) between 50% VO_2_ and 100% VO_2_ max sessions

**Fig. 2:**
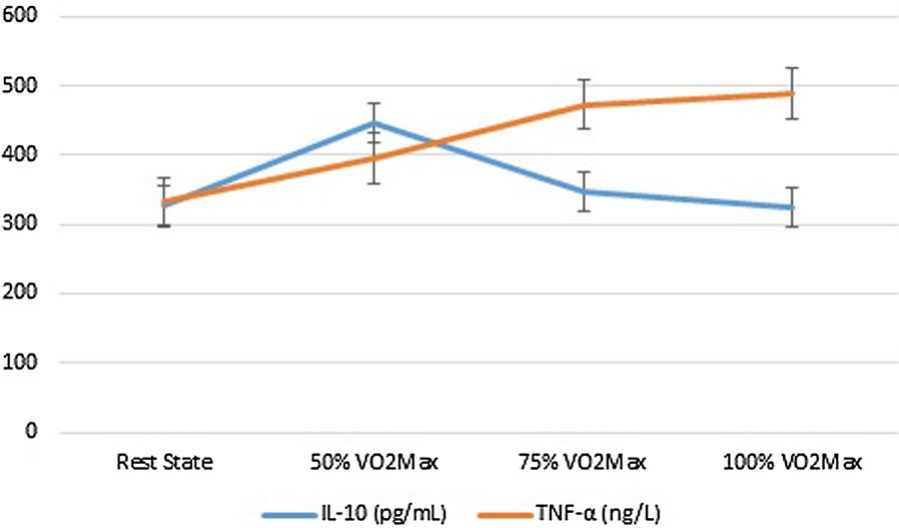
Alterations of cytokine levels in rest state and different seessions

The pairwise comparisons of biochemical values between rest state and different sessions were performed with the repeated measures ANOVA test, and the results are summarized in Table 2. Results showed significant differences in serum TNF-α levels between rest condition and 75% VO_2_ max; rest and 100% VO_2_ max session. There were significant differences in serum CRP levels between rest and 50% VO_2_ max; rest and 75% VO_2_ max sessions. There were significant differences in serum IL-10 levels between rest and 50% VO_2_ max, 50% VO_2_ max, and 100% VO_2_ max sessions. There were significant differences in serum HIF-1α levels between rest and 75% VO_2_ max session (*P* < 0.05 for all comparisons). All other comparisons were not statistically significant (*P* > 0.05 for all other pairs). The alterations in pro-inflammatory and anti-inflammatory pathways are shown in Fig. 2 with the results of IL-10 and TNF-α.

**Fig. 3:**
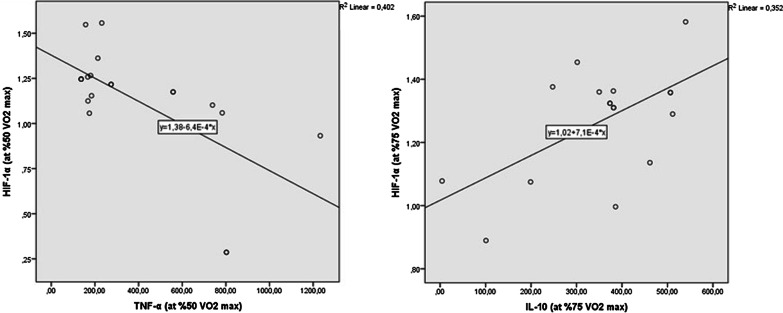
Scatter-dot graphs of Pearson correlation analysis. **A** Significant negative correlation between HIF-1α and TNF-α at 50% VO_2_ max session; (r: − 0.634, *P* = 0.003). **B** Significant positive correlation between HIF-1α and IL-10 at 75% VO_2_ max session (r: 0.593, *P* = 0.006).

Pearson correlation analyses were performed to evaluate the relationships between biochemical parameters in rest conditions and each exercise session. Results showed a high negative correlation between serum HIF-1α and TNF-α levels on 50% VO_2_ max session (r: − 0.634, *P* = 0.003). There was a moderate positive correlation between serum HIF-1α and IL-10 levels at 75% VO_2_ max session (r: 0.593, *P* = 0.006) (Fig. 3).

Correlation analysis showed that serum HIF-1α levels were negatively related to serum TNF-α levels and positively related to serum IL-10 levels. Changes in HIF-1α concentrations during exercise may have negatively affected the pro-inflammatory pathway and positively affected the anti-inflammatory pathway as a protection mechanism.

## Discussion

In this study, the acute effects of different exercise intensities on serum IL-10, TNF-α, CRP and HIF-1α levels were reported for the first time. Additionally, the study was conducted in a low altitude (1850 m) city. Exercise practice until exhaustion caused significant pro-inflammatory effects (demonstrated with TNF-α) and the optimal IL-10 response on 50% VO_2_ max decreased to nearly baseline level as the exercise intensity reached to 100% VO_2_ max. Thus, we can suggest that exercise intensity should not reach to exhaustion due to there was no improvement in the anti-inflammatory marker IL-10 and there was an increment in the pro-inflammatory marker TNF-α with the potential increase in inflammation. There is a high altitude camping center for athletes in our city, and athletes from all over the country use this center. For this reason, the study has regional and national added value.

Several studies have investigated the responses of pro-inflammatory cytokines, inflammatory cytokines and inflammatory markers to different exercise intensities and modalities, and these studies report distinct results [11, 13, 16, 17, 33, 34]. The conflicting results from previous literature may arise from differences in exercise intensities, exercise modalities, VO2 max capacities, the timing of blood sampling and biological variations.

In a study conducted with 20 soccer players with a mean age of 25.75 ± 3.99 years, participants were subjected to a single bout high high-intensity interval training and plasma IL-6, IL-1 and TNF- α levels determined before and immediately after training. There was a significant increase in plasma interleukin-6 levels after exercise; however, no significant increase in IL-1 and TNF- α levels showing an anti-inflammatory condition might occur through high-intensity interval training sessions [34]. The training protocol of this study and the exercise protocol of the current research is different, and it's known that metabolic changes may occur differently in training and exercise. However, the type of sports and age of the participants in the two studies are similar. We observed increments in both anti-inflammatory and pro-inflammatory cytokines; anti-inflammatory marker (IL-10) was increased at 50% VO_2_ session, and pro-inflammatory marker (TNF-α) was increased 75% VO_2_ and 100% VO_2_ sessions, and inflammatory (CRP) marker was increased at 50% VO_2_ and 75% VO_2_ sessions. We used high-intensity exercises, and researchers have used high-intensity interval training (HIIT). We could not show the anti-inflammatory effects of high intensity exercise in our study, although other researchers have shown the anti-inflammatory effects in their study using high-intensity interval training (HIIT).

The TNF-α level is decreased by moderate exercise (exercise intensity HRmax 60–70%) [33], and mRNA expression of TNF-α is known to be slightly elevated in skeletal muscle by endurance exercise [35]. In the previous study, a gradient increment was observed in serum TNF-α levels as exercise intensity increases. The highest TNF-α response to exercise was found at 100% VO_2_ max session when the athlete presents his maximum endurance.

A systematic review including 18 articles investigating the effects of moderate and intense exercise on inflammatory response concluded that intense long exercise protocols might activate pro-inflammatory pathways. Instead of this, moderate or high-intensity intermittent exercise protocols with suitable rest conditions may be preferred [1]. We are in line with this conclusion since we observed an inflammatory profile by determining high TNF- α and CRP levels in 75% VO_2_ max and 100% VO_2_ max sessions and optimal IL-10 concentration at 50% VO2 max session. Although there is evidence of minimal pro-inflammatory cytokine response and high anti-inflammatory cytokine release from a study conducted on athletes competing in an ironman triathlon race [36], it should be considered that triathlon race is a type of ultra-endurance exercise. We suggest IL-10 levels were not increased as expected at 75% VO2 max and 100% VO2 max exercise intensities because of pro-inflammatory effects. CRP level partially supported this suggestion, being significantly higher in the 75% VO2 max session than the rest state. Among studies investigating the impact of exercise on CRP release, most of them reveal increased CRP levels immediately after moderate [37]and intense exercise [38]. Yet, a study reports no effect of exercise modality on acute CRP response [39]. As determined the highest CRP value in 75% VO2 max session and elevated values in 75% VO2 max session compared to rest state in the present study, we can conclude that CRP partly acts together with the pro-inflammatory pathways. However, we could not determine any significant correlation between CRP and TNF- α. Although we determined optimal IL-10 levels and relatively low TNF- α levels (compared to 75% VO2 max and 100% VO2) at 50% VO2 max session, we can not recommend using this intensity to athletes since this intensity is not related to training for fitness improvements/adaptations, and as well as for soccer as the participants in the current investigation were indeed soccer players. Further, 50% intensity will not be adequate to stress the body to induce an adaptation. Acute exercise sessions lead to a complex cascade of inflammatory and pro-inflammatory pathways [40–42]. We are in line with this conclusion with altered TNF-α, IL-10, and CRP levels among sessions.

Considering the current study results and related studies, we can speculate that moderate intensity exercise with durations longer than 30 min (providing higher endurance than the present study) may be beneficial to prevent/reduce pro-inflammatory response.

It is known that disease-induced hypoxia is closely related to the activation of inflammatory pathways, but less information is available about the effects of exercise-induced hypoxia on inflammation. There is a relationship between hypoxia and the release of pro-inflammatory cytokines. Moreover, HIF-1α is important in controlling excessive inflammation [23]. Also, hypoxia, inflammation, and exercise can induce the HIF-1α pathway. It was shown that skeletal muscle HIF-1 protein content increased by 120% with hypoxia, and HIF-1α released in response to hypoxia was triggered by the effect of exercise [43]. In the present study, serum HIF-1α levels were significantly increased in 75% VO2 max session compared to rest state in a high-lander athlete population living in this location for at least 5 years.

At hypoxia conditions in the exercising person, the inflammatory pathways are regulated differently. The hypoxic and exercise stimuli are stronger in vivo than the hypoxic or inflammatory stimuli isolated in vitro [24].

Of note, when considering HIF-1α results, it should be kept in mind that high interindividual variability may be seen in the expression of HIF and its target genes in response to inflammatory or hypoxic stimuli, and single nucleotide polymorphisms (SNPs) are thought to be involved in these changes [25].

There is a relation between hypoxia and inflammation. Hypoxia can induce inflammation, and inflamed tissues may become hypoxic [20]. Limited studies investigating the effects of exercise on inflammatory pathways in hypoxic conditions revealed no changes in pro-inflammatory cytokines, and increases in anti-inflammatory cytokines, indicating the positive effects of exercising in hypoxic conditions.

The triple relation of exercise, inflammatory pathways, and oxygen consumption in a low altitude location were investigated in a previous study. HIF-1 α response is maximum on 75% VO_2_ max session and decreases from this maximum value on exhaustion. This result follows the finding that high-intensity exercise in hypoxia can further induce HIF-1α expression [43]. It is well known that high-lander athletes show better exercise performance and greater VO_2_ max capacity than sea-landers since athletes have adapted to hypoxia, and maybe some have a genetic basis, thanks to the effect of altitude [44, 45].

Although there is no agreement to define the term “high-intensity”, it widely refers to exercise intensity higher than 75% VO_2_ max [23]. A speculative model suggests that HIF-1α and PGC-1α act as mediators in the adaptation of skeletal muscle. The mediators lead to upregulation of mitochondrial biogenesis, angiogenesis via activation of VEGF and a shift in the skeletal muscle fibre type. Both high-intensity exercise/training and hypoxia lead to this mechanism to upregulate skeletal muscle adaptation [23].

We observed the optimum HIF-1 α response in a 75% VO2 max session in the present study.

HIF-1 α response did not increase when the exercise intensity was reached from 75% VO_2_ max to 100% VO_2_ max in the present study. We attribute this because the athletes have developed a physiological adaptation to hypoxia thanks to living at low altitudes. Correlation analyses revealed a high negative correlation between serum HIF-1α and TNF-α levels on 50% VO_2_ max session (r: − 0.634, *P* = 0.003) and a moderate positive correlation between serum HIF-1α and IL-10 levels at 75% VO_2_ max session (r: 0.593, *P* = 0.006). Results suggest that increased HIF-1α levels reflect the pro-inflammatory condition in 50% VO_2_ max session and the anti-inflammatory condition in 75% VO_2_ max session. We determined maximum TNF-α response and similar IL-10 response compared to baseline in 100% VO_2_ max session. We can conclude that the pro-inflammatory effects of hypoxia and anti-inflammatory effects of the exercise was probably due to activating the release of anti-inflammatory cytokines and downregulating toll-like receptor (TLR) signalling [23].

Studies with different exercise protocols have shown that high-intensity exercise (above 75% of the peak power output) provides similar or even higher benefits than a low-intensity continuous exercise in improving heart health, respiratory health, and metabolic health. Increases in peak power outputs during exercise result in increased metabolic responses, compromising skeletal muscle integrity, which can cause early onset of fatigue and exhaustion. Therefore, the selection of exercise intensity should be made carefully to avoid undesirable consequences. Taken together, TNF-α, IL-10, CRP, and HIF-1α results, we again suggest that exercise intensity should not reach to exhaustion. Despite its originality, the current study has a limitation. It would be better ELISA results should be supported with western blotting analysis and mRNA expression levels of proteins.

## Conclusions

There is a tight connection between hypoxia and inflammation, and studies investigating the effects of exercise intensity in hypoxic and inflammatory pathways are limited. There is no available study in any athletic population reporting the acute changes on serum IL-10, TNF-α, CRP and HIF-1α levels induced by different exercise intensities. We noted that both inflammatory and anti-inflammatory pathway is induced on different exercise intensities. As the need for oxygen increases, the inflammatory pathway (by TNF- α and CRP) is induced, and anti-inflammatory cytokine IL-10 reaches optimal value on exercise intensity of 50% VO_2_ max. Exercise regimens (not reached to exhaustion) are recommended to prevent inflammation, hypoxia-induced damage, and existing muscle damage progression if any. Further studies on different athlete groups should be conducted to determine the optimum exercise intensity and maximum benefit.

## Data Availability

The datasets used and/or analyzed during the current study are available from the corresponding author on reasonable request.

## Abbreviations

VO_2_ max: Maximal oxygen consumption values
TNF-α: Tumor necrosis factor alpha
IL-10: Interleukin-10
CRP: C-reactive protein
HIF-1 α: Hypoxia inducible factor-1 alpha
